# No ICU Unless 2.0 – Refining ICU admission criteria after elective craniotomy

**DOI:** 10.1016/j.bas.2025.105878

**Published:** 2025-11-17

**Authors:** Lina-Elisabeth Qasem, Christian Seemann, Frenki Shima, Dilara Karakaya, Felix Corr, Jan Oros, Celina Ufken, Jan-Hendrik Schröder, Ali Al-Hilou, Stefanos Voglis, Jürgen Konczalla, Michael Eibach, Kristin Lucia, Kai Zacharowski, Ulrich Strouhal, Christian Grefkes, Daniel Jussen, Vincent Prinz, Marcus Czabanka

**Affiliations:** a Goethe University Frankfurt, University Hospital, Department of Neurosurgery, Frankfurt Am Main, Germany; b Department of Neurosurgery, University Hospital Zurich, Zurich, Switzerland; c Goethe University Frankfurt, University Hospital, Department of Anaesthesiology, Intensive Care Medicine & Pain Therapy, Frankfurt Am Main, Germany; d Goethe University Frankfurt, University Hospital, Department of Neurology, Frankfurt Am Main, Germany

**Keywords:** Intensive care medicine, Intensive care unit, Postoperative monitoring, Elective craniotomy, Risk stratification

## Abstract

**Objective:**

Routine intensive care unit (ICU) admission following elective craniotomy is still standard practice in most neurosurgical centers, despite growing evidence supporting selective monitoring strategies.

**Research question:**

We aimed to refine ICU admission criteria by identifying predictors of ultimately required postoperative ICU transfers and by developing a pragmatic risk score.

**Material and methods:**

We retrospectively analyzed adult patients who underwent elective craniotomy from 2021 to 2024. Patients were divided into two cohorts: Cohort A included patients transferred to the general ward as planned, while Cohort B included those requiring unplanned ICU admission after initial general ward assignment. Baseline and surgical characteristics, complication rates and outcome parameters were compared. Univariate and multivariable logistic regression were performed to identify predictors of ultimately required ICU admission. A risk score was developed based on significant predictors.

**Results:**

Of 1441 patients undergoing elective craniotomy, 1162 (81 %) were preoperatively scheduled for postoperative care on the general ward. Of these, 153 patients (13 %) required unplanned ICU admission. Analysis identified independent risk factors: surgical time >143 min (OR 4.73; p < 0.0001), blood loss >500 mL (OR 1.89; p = 0.0098), age >72 years (OR 2.18; p = 0.0037), and intraventricular lesion location (OR 16.92; p < 0.0001). A 3-point risk score was constructed, with ICU admission rates increasing from 2.5 % (0 points) to 36 % (3 points).

**Discussion and conclusions:**

Selective ICU admission after elective craniotomy appears safe when guided by risk assessment. The score may support clinical decision-making, reduce unnecessary ICU utilization, and promote resource-efficient postoperative care – even in complex or infratentorial cases.

## Highlights

• Surgical time, blood loss, age and intraventricular lesions as independent factors.
• A risk score was developed to guide ICU/IMC triage after elective craniotomy.
• Intraventricular lesion location showed the strongest association with ICU need.
• The data support safe postoperative management outside the ICU.
• Selective ICU use may reduce overtreatment and improve resource allocation.

## 1 Introduction

While routine postoperative intensive care unit (ICU) management following elective craniotomy remains standard practice in most neurosurgical centers worldwide (Hecht et al., 2014; Awad, 2014), increasing attention has been directed toward alternative strategies, including selective ICU admission and postoperative monitoring on regular neurosurgical wards (Qasem et al., 2022; Florman et al., 2017; Beauregard and Friedman, 2003). Some institutions have even reported successful same-day discharge in carefully selected patients (Goettel et al., 2014; Carrabba et al., 2008; Grundy et al., 2008). Currently, evidence does not allow defining standardized protocols to guide ICU admission and postoperative monitoring.

The primary rationale for postoperative ICU monitoring is the early detection of potentially life-threatening complications such as postoperative hemorrhage, cerebral edema, ischemic stroke, seizures or hydrocephalus – events that may cause neurological deterioration or require urgent revision surgery (Reponen et al., 2015). Postoperative hemorrhage occurs in approximately 1–4 % of patients undergoing craniotomy and most often leads to clinical deterioration within the first 2–6 h after surgery (Taylor and Wellings, 1995; Lassen et al., 2011). Beyond this window, acute complications are less frequent and can typically be recognized through routine neurological assessments on regular wards (Qasem et al., 2022; Lonjaret et al., 2017; Laan et al., 2020).

Growing evidence suggests that most neurosurgical patients do not require ICU-level intervention postoperatively and that routine ICU admission may result in unnecessary resource utilization without improving patient outcomes (Qasem et al., 2022; Florman et al., 2017; Beauregard and Friedman, 2003; Bui et al., 2011; Hanak et al., 2014; Badenes et al., 2017). In recent years, several institutions have proposed more selective strategies for postoperative ICU admission based on clinical risk factors (Neumann et al., 2023; Munari et al., 2022; Cinotti et al., 2018; Herrero et al., 2017). Approaches such as the “No ICU Unless” protocol (Qasem et al., 2022; Laan et al., 2020) advocate for postoperative transfer to a general neurosurgical ward unless predefined neurological, neurosurgical, or anesthesiologic criteria are met. At our department, we implemented such a strategy in 2021 with the following “No ICU Unless” criteria:

(1) **Neurological or neurosurgical criteria**: posterior fossa tumors >3 cm in diameter; involvement of lower cranial nerves with (potential for) dysphagia and aspiration; or altered level of consciousness prior to surgery.
(2) **Anesthesiologic criteria**: cardiopulmonary or hemodynamic risk factors as assessed by the attending anesthesiologist, including an American Society of Anesthesiologists (ASA) score of ≥4, coagulation disorders, or anticipated difficult airway management.

However, in clinical practice, we observed that ICU admission was frequently triggered by the presence of a single criterion, often resulting in unnecessary ICU monitoring, even in otherwise stable patients. This led to a progressive softening of the initial strategy, with more patients ultimately transferred to the general ward despite fulfilling one of the predefined criteria.

The aim of the present study was to reassess and refine current ICU admission criteria using data from a large, contemporary cohort of patients undergoing elective craniotomy. Specifically, we sought to identify predictors of unplanned postoperative ICU or intermediate care unit (IMC) admission and to develop a pragmatic risk score to support clinical decision-making.

## 2 Methods and material

**Study design:** We conducted a retrospective, single-center cohort study including all adult patients who underwent elective craniotomy at the Department of Neurosurgery, University Hospital Frankfurt, Germany between May 2021 and December 2024. Emergency procedures were excluded from the analysis. The primary outcome was unplanned postoperative admission to an ICU or IMC after patients were initially scheduled for standard postoperative care on the general ward, following a 2–4 h observation period in the recovery unit.

Patients who were ultimately scheduled for postoperative ICU admission included:

- Patients with altered level of consciousness prior to surgery: GCS ≤14
- Patients undergoing highly complex procedures e.g., bypass surgery, large vestibularis schwannomas with consecutive hydrocephalus (Koos Grade IV), giant olfactory groove meningiomas
- Patients with an indication for preoperative external ventricular drain (EVD) insertion
- Patients with a combination of advanced age and significant comorbidities as determined by the attending anesthesiologist:
  ⁃ ASA ≥4
  ⁃ Obstructive sleep apnea syndrome
  ⁃ Cardiopulmonary or hemodynamic risk factors
  ⁃ Difficult airways
  ⁃ Coagulation disorders

Patient characteristics, comorbidities, lesion location and diagnosis group (tumor, epilepsy, vascular or infection), surgical procedures and parameters, complications and postoperative outcomes were extracted from the institutional electronic health record. Preoperative functional status was assessed using modified rankin Scale scores (mRS) and Karnofsky Performance Scale scores (KPS), and anesthesiologic risk was graded using the ASA classification.

Surgical parameters included surgical duration (defined as skin-to-skin time), patient positioning (prone, lateral or supine) and intraoperative blood loss (as documented in the anesthesia protocol).

Patients were divided into two cohorts based on their actual postoperative care setting:

- **Cohort A** included patients who were preoperatively scheduled for transfer to the general ward and were transferred to the general ward as planned.
- **Cohort B** included patients who were preoperatively scheduled for transfer to the general ward but ultimately required unplanned admission to the ICU or IMC postoperatively.

Patients preoperatively scheduled for postoperative ICU/IMC admission were excluded from the analysis.

Approval from the local ethics committee was obtained prior to the study (No. 2025–2376). This research was conducted in accordance with the Declaration of Helsinki and all relevant guidelines and regulations. As the study involves retrospective analysis, a waiver of informed consent was granted by the Institutional Review Board (IRB) of Goethe University Frankfurt am Main, Germany, where broad consent includes patient consent for retrospective data analyses. Routine surgical consent was obtained prior to surgery.

Statistical analysis: Data were analyzed using GraphPad Prism (version 9.5.1; GraphPad Software, San Diego, CA, USA) and JASP (version 0.19.3, JASP Team, University of Amsterdam). Descriptive statistics were calculated for all variables, with continuous data presented as means with standard deviations (SD) or medians with ranges or interquartile range (IQR), as appropriate. The normality of data distribution was assessed using the Kolmogorov-Smirnov test. Comparisons between the two cohorts were conducted using the Mann-Whitney *U* test for nonparametric continuous data and the unpaired *t*-test for parametric continuous data. Pearson's Chi-squared test was used for categorical data. Univariate and multivariable logistic regression analyses were performed to identify independent predictors of unplanned ICU/IMC admission. To avoid confounding by non-clinical factors, only patients with medically indicated unplanned ICU/IMC admissions were included in the univariate and multivariable regression analyses; cases with non-medical or capacity-related admission reasons were excluded.

For continuous variables, optimal cutoff values were determined using receiver operating characteristic (ROC) curve analysis, with the Youden Index applied to identify the point of maximal combined sensitivity and specificity. Based on the multivariable model, a simplified clinical risk score was constructed. The predictive accuracy of the score was evaluated using ROC analysis, and the area under the curve (AUC) was calculated with corresponding 95 % confidence intervals (CI). ICU/IMC admission rates were assessed across score categories to evaluate calibration and clinical applicability. A p-value <0.05 was considered statistically significant. Missing data were rare (<5 % across all variables). We conducted a complete case analysis and did not perform imputation.

## 3 Results

### 3.1 Postoperative transfers

Between May 2021 and December 2024, a total of 1441 elective craniotomies were performed at the Department of Neurosurgery, University Hospital Frankfurt, Germany. Of these, 1162 patients (81 %) were scheduled for postoperative transfer to the general ward following a 2–4-h observation period in the recovery room. The remaining 279 patients (19 %) were assigned to postoperative monitoring in the ICU or IMC based on predefined criteria (see Methods section) and were excluded from the analysis.

**Fig. 1:**
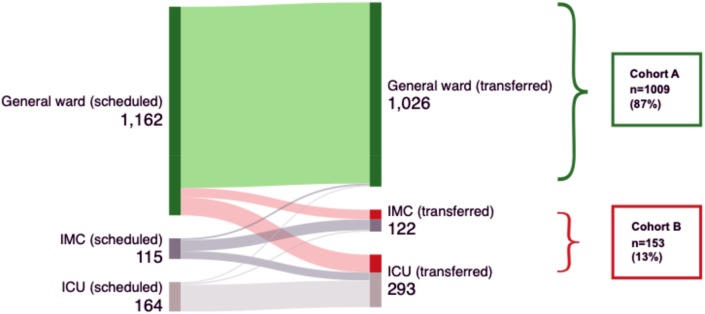
Postoperative patient transfers.

Among those scheduled for general ward transfer, 1009 patients (87 %) were transferred as planned (Cohort A), while 100 patients (8.5 %) required unplanned admission to the ICU, and 53 patients (4.5 %) were transferred to the IMC unplanned (Cohort B). Fig. 1 provides a visual overview of postoperative patient transfers.

### 3.2 Cohort A: Planned transfer to the general ward

Baseline characteristics: A total of 1009 patients (87 %) were transferred to the general ward after elective craniotomy as preoperatively planned. This cohort included 456 women (45 %) and 556 men (55 %), with a median age of 57 (IQR, 46–66) years. The majority of patients (82 %) were treated for brain tumors, including gliomas, meningiomas, skull base tumors, and metastases. Other diagnoses included vascular pathologies (11 %) such as aneurysms, vascular malformations, epilepsy (3.5 %), infectious diseases (1 %) and miscellaneous conditions (2.5 %) including cerebrospinal fluid (CSF) fistulas and colloid cysts. Lesions were predominantly supratentorial (88 %), with 124 patients (12 %) presenting with infratentorial lesions, intraventricular lesions in nine cases (1 %), cerebellopontine angle (CPA) lesions in 54 cases (5 %), and skull base lesions in 88 cases (9 %).

**Fig. 2:**
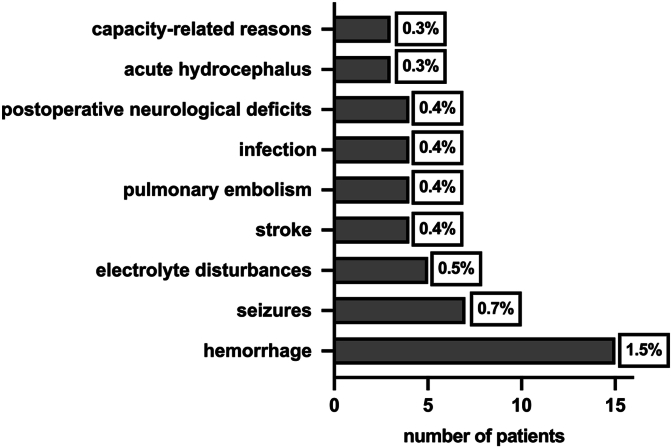
Reasons for readmission to the ICU/IMC from the general ward in Cohort A.

Re-admission to the ICU/IMC after initial postoperative transfer to the normal ward: Forty-seven patients (4.7 %) required secondary admission to the ICU/IMC following their initial postoperative transfer to the general ward. Re-admission occurred on average on postoperative day two (range, 0–12), primarily due to complications such as hemorrhage (15 cases; 1.5 %), seizures (7 cases; 0.7 %), stroke (4 cases; 0.4 %), pulmonary embolism (4 cases; 0.4 %), infections or electrolyte disturbances (7 cases; 0.7 %), and new postoperative neurological deficits (4 cases; 0.4 %). Three patients (0.3 %) underwent surgical intervention and subsequent ICU monitoring due to acute hydrocephalus. An additional three patients (0.3 %) were re-admitted for non-medical, capacity-related reasons. No deaths in Cohort A were attributable to postoperative complications during ward-based care. Fig. 2 illustrates the indications for ICU/IMC re-admission from the general ward in Cohort A.

Complications: Major complications occurred in 41 patients in Cohort A (4 %) and included hemorrhage (23 cases; 2 %), postoperative edema (4 cases; 0.4 %), hydrocephalus (6 cases; 0.6 %), stroke (4 cases; 0.4 %), and CSF leakage (4 cases; 0.4 %). Among hemorrhages, 18 patients (1.8 %) required surgical evacuation or EVD placement, typically on postoperative day one (range 0–3). Edema led to decompressive surgery or EVD placement in two patients. Of the hydrocephalus cases, three required EVD or ventriculoperitoneal (VP) shunt placement. CSF leakage was managed surgically (n = 1), via VP shunting (n = 1), or lumbar drainage (n = 2). Minor complications included pneumonia (1.5 %), pulmonary embolism (0.7 %), seizures (2 %), and documented delirium (3.5 %).

This section summarizes major and minor postoperative complications, regardless of whether ICU/IMC admission was required for their management. Several of these complications were successfully managed on the general ward.

Clinical outcomes: The median preoperative mRS score was 1 (IQR, 1–2), remaining 1 (IQR, 0–6) postoperatively. Brain tumor patients presented with a median preoperative KPS of 90 % (IQR, 80–90) and median postoperative KPS of 90 % (IQR, 80–100). Patients spent an average of 0.5 days (±3) in the ICU or IMC. Emergency cranial imaging was performed in 82 patients (8 %) prior to routine postoperative imaging.

### 3.3 Cohort B: Unplanned postoperative admission to the ICU/IMC

Baseline characteristics: A total of 153 patients (13 %) required unplanned ICU/IMC admission immediately after surgery despite initial ward assignment. This cohort included 75 women (49 %) and 78 men (51 %), with a median age of 59 (IQR, 48–71) years. The majority of patients (84 %) were treated for brain tumors, including gliomas, meningiomas, skull base tumors, and metastases. Other diagnoses included vascular pathologies (8 %) such as aneurysms, vascular malformations, epilepsy (5 %), infectious diseases (0.6 %) and miscellaneous conditions (3 %) including CSF fistulas and colloid cysts. Lesions were predominantly supratentorial (86 %), with 22 patients (14 %) presenting with infratentorial lesions, intraventricular lesions in six cases (4 %), CPA lesions in 12 cases (8 %), and skull base lesions in 18 cases (12 %).

**Fig. 3:**
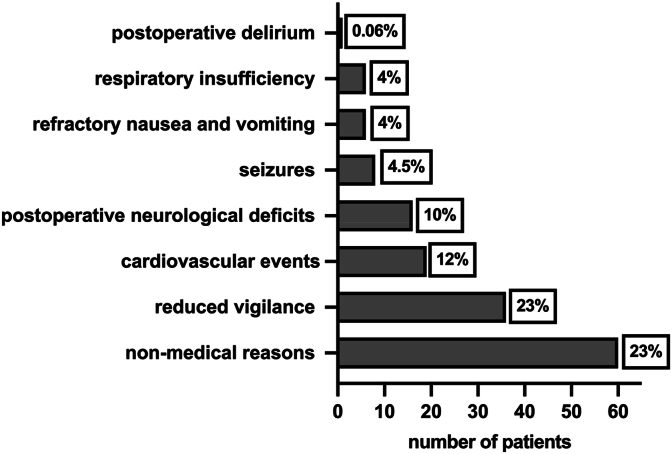
Reasons for unplanned ICU/IMC admission in Cohort B.

Indications for unplanned ICU/IMC admission: Non-medical reasons accounted for 60 of 153 cases (39 %) of unplanned admissions, mostly due to bed shortages or staffing constraints. Among medical indications, 35 patients (23 %) were admitted due to reduced postoperative vigilance, while intraoperative cardiac arrhythmias or circulatory dysregulation were observed in 18 patients (12 %), including three cases of severe blood loss (>3 L) requiring vasopressor support. Six patients (4 %) were admitted for postoperative respiratory insufficiency, 17 patients (10 %) for significant new neurological deficits requiring intensified care, and nine patients (6 %) for refractory nausea and vomiting. Seizures occurred intra- or postoperatively in seven patients (4.5 %). One patient (0.06 %) developed severe postoperative delirium necessitating IMC care. Fig. 3 visualizes postoperative unplanned ICU/IMC admissions.

Complications: Major complications occurred in 23 patients in Cohort B (15 %) and included hemorrhage (n = 11; 7 %), edema (n = 4; 3 %), hydrocephalus (n = 2; 1.3 %), stroke (n = 4; 3 %), hygroma (n = 1; 0.7 %), CSF fistula (n = 1; 0.7 %), and respiratory failure (n = 4; 3 %). Of the hemorrhages, 10 patients required surgical evacuation, while one case in a glioblastoma patient resulted in palliative care. Edema necessitated decompressive surgery in one case. Stroke was managed conservatively in four cases. Hydrocephalus required EVD placement in two cases. Respiratory failure was attributable to pneumonia (n = 3) or pleural effusion (n = 1). Minor complications included pneumonia (5 %), pulmonary embolism (2 %), seizures (5 %), and delirium (13 %).

Clinical outcomes: The median preoperative mRS score was 1 (IQR, 1–2), increasing to 1.5 (IQR, 1–3) postoperatively. Brain tumor patients presented with a median preoperative KPS of 90 % (IQR, 70–90) and median postoperative KPS of 80 % (IQR, 60–100). Patients spent an average of 4 days (±6) in the ICU or IMC. Emergency cranial imaging was performed in 57 patients (37 %) prior to routine postoperative imaging.

### 3.4 Comparison of Cohort And Cohort B

Baseline parameters: The median age was higher in Cohort B compared to Cohort A (59 vs. 57 years, p = 0.024). While sex distribution and most comorbidities were comparable between cohorts, arterial hypertension (37 % vs. 29 %; p = 0.049) and diabetes mellitus (16 % vs. 10 %; p = 0.015) occurred significantly more often in Cohort B. The distribution of surgical diagnoses and lesion locations was broadly similar, though Cohort B included more intraventricular lesions (4 % vs. 0.9 %; p = 0.007).

Surgical parameters: The comparison of surgical parameters, including surgical time, intraoperative blood loss, and ASA score, revealed highly significant differences between the cohorts. Surgical time was significantly longer in Cohort B (177 [95 % CI, 165–188] minutes vs. 127 [95 % CI, 124–130] minutes; p < 0.0001), and intraoperative blood loss was substantially higher in Cohort B (692 [95 % CI, 435–948] mL vs. 318 [95 % CI, 294–341] mL; p < 0.0001). Although the median ASA score was three in both cohorts, a statistically significant difference was observed in the overall distribution (p = 0.0049), suggesting a shift in distribution toward higher ASA classifications among patients requiring unplanned ICU or IMC admission.

Outcome parameters: Complication rates were markedly higher in Cohort B. Major complications occurred in 15 % vs. 4 %, and minor complications in 26 % vs. 8 % (both p < 0.0001). Cohort B had longer ICU/IMC stays (4 [95 % CI, 2.3–5] days vs. 0.5 [95 % CI, 0.2–0.5] days; p < 0.0001) and a greater proportion of emergency cranial imaging (37 % vs. 8 %, p < 0.0001).

Although the preoperative mRS was 1 in both cohorts, statistical analysis revealed a highly significant difference in the overall distribution (p < 0.0001), indicating a greater functional impairment among patients requiring unplanned ICU/IMC admission. When dichotomized into favorable (mRS 1–2) and unfavorable (mRS 3–6) preoperative status, patients with unfavorable scores demonstrated a 1.56-fold increased odds of unplanned ICU/IMC admission (95 % CI, 0.96–2.54; p = 0.0757). A similar observation was made in patients with brain tumors: Although median pre- and postoperative KPS scores were comparable between cohorts, statistical analysis revealed a significant difference in score distribution (p < 0.0001, respectively). See Table 1 for baseline, intraoperative, and outcome-related differences between Cohort A and Cohort B.

**Table 1:** Comparison of Cohort A and Cohort B with respect to baseline characteristics, surgical parameters, and clinical outcomes.

		unscheduled ICU/IMC admission		
characteristic	overall	no (Cohort A)	yes (Cohort B)	p-value
**patients, n (%)**	1162 (100 %)	1009 (87 %)	153 (13 %)	
female, n (%)	630 (54 %)	456 (45 %)	75 (49 %)	
age, median (IQR)	58 (47–67)	57 (46–66)	59 (48–71)	0.0241^b^
**pre-existing conditions**				
arterial hypertension, n (%)	350 (30 %)	293 (29 %)	57 (37 %)	**0.049**^c^
malignancy, n (%)	261 (25 %)	232 (23 %)	29 (19 %)	0.312^c^
diabetes mellitus, n (%)	125 (11 %)	101 (10 %)	24 (16 %)	**0.015**^c^
hypothyroidism, n (%)	171 (15 %)	151 (15 %)	20 (13 %)	0.622^c^
anticoagulation, n (%)	69 (6 %)	60 (6 %)	9 (6 %)	1.000^c^
cardiac disease, n (%)	75 (6 %)	61 (6 %)	14 (9 %)	0.201^c^
**diagnosis group**				
brain tumors, n (%)	955 (82 %)	827 (82 %)	128 (84 %)	0.691^c^
vascular pathologies, n (%)	123 (11 %)	110 (11 %)	12 (8 %)	0.313^c^
epilepsy, n (%)	43 (4 %)	35 (3.5 %)	8 (5 %)	0.398^c^
infectious diseases, n (%)	11 (0.9 %)	10 (1 %)	1 (0.6 %)	1.000^c^
other diagnoses, n (%)	30 (3 %)	25 (2.5 %)	5 (3 %)	0.764^c^
**lesion location**				
supratentorial, n (%)	1019 (88 %)	888 (88 %)	132 (86 %)	0.633^c^
infratentorial, n (%)	143 (12 %)	121 (12 %)	22 (14 %)	0.481^c^
intraventricular, n (%)	15 (1.3 %)	9 (0.9 %)	6 (4 %)	**0.007**^c^
CPA, n (%)	62 (5 %)	54 (5 %)	12 (8 %)	0.292^c^
skull base, n (%)	109 (9 %)	88 (9 %)	18 (12 %)	0.286^c^
**positioning**				
supine position, n (%)	883 (76 %)	767 (76 %)	116 (76 %)	1.000^c^
prone position, n (%)	142 (12 %)	121 (12 %)	21 (14 %)	0.633^c^
lateral position, n (%)	136 (12 %)	121 (12 %)	15 (10 %)	0.516^c^
**surgical characteristics**				
surgical time in minutes, mean (SD)	134 (±55)	127 (±50)	177 (±78)	**<0.0001**^a^
blood loss in mL, mean (SD)	273 (±605)	318 (±318)	692 (±152)	**<0.0001**^a^
ASA score, median (IQR)	3 (2–3)	3 (2–3)	3 (2–3)	**0.0049**^b^
**clinical outcome**				
major complications, n (%)	64 (5 %)	41 (4 %)	23 (15 %)	**<0.0001**^c^
minor complications, n (%)	117 (10 %)	77 (8 %)	40 (26 %)	**<0.0001**^c^
preop mRS, median (IQR)	1 (1–2)	1 (1–2)	1 (1–2)	**<0.0001**^b^
postop mRS, median (IQR)	1 (0–6)	1 (0–6)	1.5 (1–3)	**<0.0001**^b^
preop KPS, median (IQR)	90 (70–90)	90 (80–90)	90 (70–90)	**<0.0001**^b^
postop KPS, median (IQR)	90 (60–100)	90 (80–100)	80 (60–100)	**<0.0001**^b^
ICU/IMC stay in days, mean (SD)	0.8 (±3)	0.6 (±3)	4.1 (±6)	**<0.0001**^a^

a *t*-Test.

b Wilcoxon rank-sum test.

c Pearson's Chi-squared test; CPA, cerebellar pontine angle; IQR, interquartile range; KPS, Karnofsky Performance Scale; SD, standard deviation.

### 3.5 Risk modeling

To ensure model validity, only immediate postoperative ICU/IMC admissions on the day of surgery (Cohort B) were included in the regression analyses. Late ICU/IMC re-admissions (n = 47 of Cohort A) were analyzed descriptively but were not part of the predictive modeling. In addition, to avoid bias, patients admitted to the ICU/IMC for non-medical reasons (n = 60) were excluded from all logistic regression analyses.

**Fig. 4:**
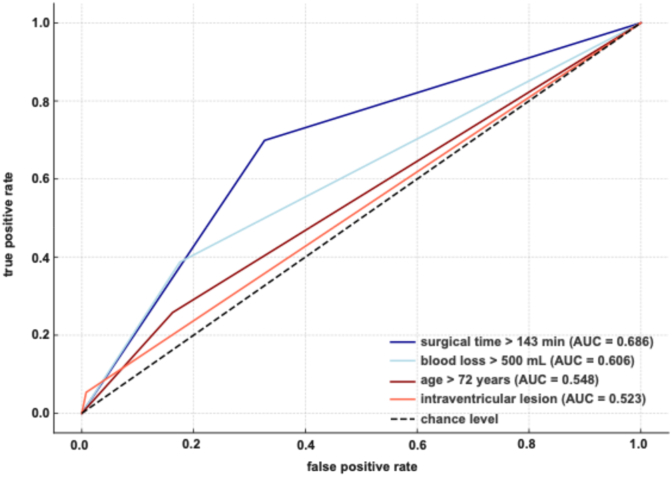
ROC curves for independent risk factors.

Univariate logistic regression identified four significant risk factors for unplanned ICU/IMC admission: surgical duration ≥143 min (OR 4.4; 95 % CI 2.68–7.21; p < 0.0001), blood loss ≥500 mL (OR 2.33; 95 % CI 1.39–3.90; p = 0.0001), age ≥72 years (OR 1.76; 95 % CI 1.0–3.10; p = 0.0037), and intraventricular lesion location (OR 15.93; 95 % CI 4.72–53.73; p = 0.0007). ROC curve analysis (Fig. 4) showed surgical duration had the strongest discriminatory value (AUC 0.686), followed by blood loss (AUC 0.606), age (AUC 0.548), and intraventricular lesion (AUC 0.523).

Multivariable logistic regression confirmed these four as independent predictors: surgical time (OR 4.73; 95 % CI 2.86–7.84; p < 0.0001), blood loss (OR 1.89; 95 % CI 1.17–3.06; p = 0.0098), age (OR 2.18; 95 % CI 1.29–3.68; p = 0.0037), and intraventricular location (OR 16.92; 95 % CI 4.97–57.59; p < 0.0001). The model had strong discriminative capacity (AUC 0.86) and achieved a sensitivity of 76.3 % and specificity of 68.5 % at a Youden-optimized threshold.

Infratentorial lesion location was not significantly associated with an increased risk of unplanned ICU/IMC admission (OR, 0.81; 95 % CI, 0.50–1.32; p = 0.403). Although the odds ratio was below 1, suggesting a possible trend, this association did not reach statistical significance. With the exception of intraventricular lesions, no other anatomical location was significantly associated with elevated risk. Diagnosis groups were not significantly associated with unplanned ICU/IMC admission in the multivariable regression model.

A simplified 3-point clinical risk score was developed using the three generalizable predictors identified in multivariable logistic regression: surgical time >143 min, blood loss >500 mL, and age >72 years. Intraventricular lesions were excluded from the clinical risk score due to their limited applicability and poor individual discriminatory power (AUC 0.52).

**Fig. 5:**
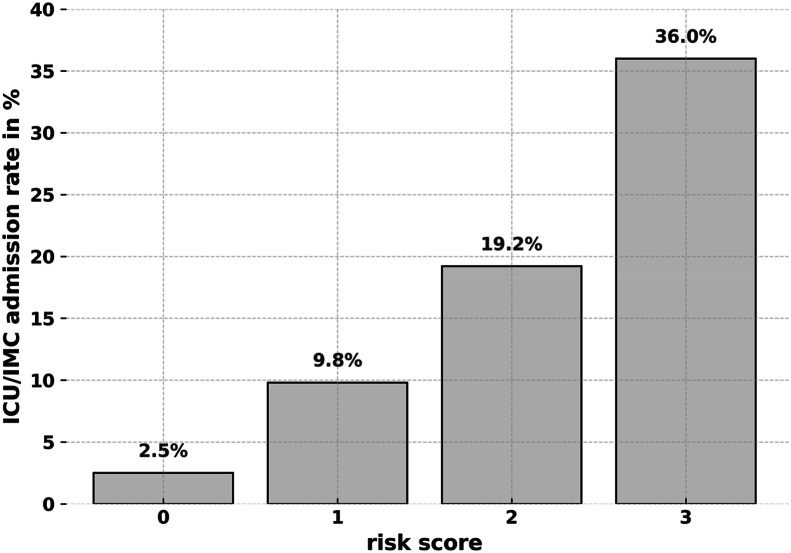
ICU/IMC admission rate by clinical risk score.

**Fig. 6:**
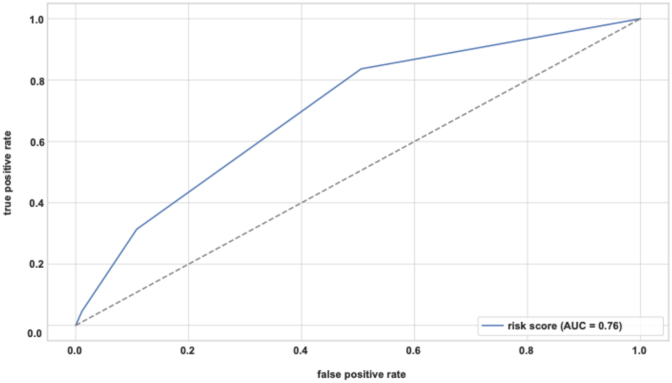
ROC curve risk score.

ICU/IMC admission rates increased stepwise with the risk score: 2.5 % (0 points), 9.8 % (1 point), 19.2 % (2 points), and 36 % (3 points). No patient presented with all four original risk factors. The final score demonstrated moderate discriminative ability (AUC 0.76). See Fig. 5, Fig. 6 for details.

## 4 Discussion

In this retrospective analysis of 1162 patients undergoing elective craniotomy at a tertiary neurosurgical center, we aimed to refine and optimize the selective postoperative ICU admission strategy known as the “No ICU Unless” (Qasem et al., 2022; Laan et al., 2020) approach. Our findings demonstrate that the majority of patients (>80 %) could be safely managed without routine intensive care monitoring, with only 13 % requiring unplanned transfer to an ICU or IMC. These unplanned admissions were associated with longer surgical duration, greater intraoperative blood loss, advanced age, and intraventricular lesion location. Based on these factors, we developed a pragmatic 3-point risk score to support postoperative risk stratification.

### 4.1 Prediction scores and ICU criteria

Neumann et al. (2023) recently evaluated two existing prediction scores (Munari et al., 2022; Cinotti et al., 2018) for postoperative ICU triage after brain tumor surgery. While both models included clinically relevant parameters, their predictive performance was limited (AUCs of 0.65 and 0.67^17^). Moreover, these and most previously published scores (Florman et al., 2017; Laan et al., 2020; Neumann et al., 2023; Franko et al., 2018) were developed exclusively in patients undergoing supratentorial tumor resections, limiting their broader applicability.

In contrast, our score was derived from a heterogeneous cohort including all cranial pathologies. While the final 3-point score demonstrated moderate predictive capacity (AUC 0.76), it should be interpreted as a pragmatic, rather than high-precision, triage tool. A score of 3 was associated with an ICU/IMC admission rate of 36 % and may serve as a practical threshold to prompt early ICU or IMC bed planning during surgery. Patients with a score of 0–2 may generally be managed safely on the ward.

Although intraventricular tumor location had the highest odds ratio in our multivariable analysis (OR ∼17), its low AUC (0.52) suggests limited contribution to overall model discrimination, indicating a strong but infrequent predictor within a specific subgroup. Although infratentorial lesions have traditionally been considered an indication for routine postoperative ICU monitoring due to presumed risks such as edema and early hydrocephalus (Lassen et al., 2011; Lonjaret et al., 2017; Steinruecke et al., 2023), our study demonstrates that postoperative management outside the ICU is feasible even in this subgroup. In multivariable analysis, neither infratentorial location (OR, 0.81; 95 % CI, 0.50–1.32; p = 0.403) nor cerebellopontine angle lesions (OR, 1.36; 95 % CI, 1.99–14.90; p = 0.375) were independent predictors of unplanned ICU or IMC admission. These findings support extending a risk-adapted ICU admission strategy to patients with infratentorial pathology. Table 2 summarizes previously published scoring systems and ICU triage criteria.

**Table 2:** Existing score and ICU criteria.

study	scope	cohort	AUC	score composition/ICU criteria
Cinotti et al. (2018) (Cinotti et al., 2018), CranioScore	brain tumors	multi center	0.65^17^	*score:* pre OP GCS <14, history of brain tumor surgery, greatest tumor diameter, midline shift, transfusion of blood products, maximum and minimal systolic arterial pressure, duration of surgery
Munari et al. (2022) (Munari et al., 2022)	brain tumors	single center	0.67^17^	*score:* ASA, duration of surgery, blood loss, tumor volume
Florman et al. (2017) (Florman et al., 2017)	supratentorial brain tumors	single center	0.67	*ICU criteria:* seizures, intracranial drains, need for transfusion, coagulopathy, diabetes insipidus, need for intravenous medication drips at the time of transfer
Laan et al. (2020) (Laan et al., 2020)	supratentorial brain tumors	single center	not reported	*ICU criteria:* duration of surgery, comorbidities, blood loss, functional status
Franko et al. (2018) (Franko et al., 2018)	supratentorial brain tumors	single center	0.72	*score:* KPS <70, general endotracheal anesthesia, early postoperative complications
No ICU Unless 2.0 (present study)	all elective craniotomies	large single center	0.76	*score:* duration of surgery (>143 min), blood loss >500 mL, intraventricular location, age >72

Abbreviations: ASA, american association of anesthesiologists classification; AUC, area under the receiver operating characteristic curve; GSC, glasgow coma scale; ICU, intensive care unit; KPS, karnofsky performance scale.

### 4.2 Monitoring versus actual treatment

Furthermore, the overall postoperative complication rate among patients managed outside the ICU was low, and only 4.7 % required delayed transfer to a monitored setting. These findings support the concept that a risk-adapted ICU admission strategy can reduce unnecessary ICU utilization without compromising patient safety. Routine invasive ICU monitoring – such as electrocardiography, arterial blood pressure monitoring, or strict fluid balance charting – has limited utility in detecting intracranial complications and does not prevent adverse neurological outcomes (Qasem et al., 2022; Bui et al., 2011). In a prior institutional analysis, only 11 % of ICU patients after elective craniotomy required any specific therapeutic intervention (Qasem et al., 2022). Several patient-related factors have been associated with an increased likelihood of ICU treatment, including diabetes mellitus, advanced age, prolonged surgical duration, large intraoperative blood loss, and higher anesthetic risk (ASA classification) (Lonjaret et al., 2017; Bui et al., 2011; Hanak et al., 2014; Badenes et al., 2017). Nonetheless, the majority of ICU-managed patients receive monitoring alone without the need for therapeutic intervention (Qasem et al., 2022; Florman et al., 2017; Beauregard and Friedman, 2003; Badenes et al., 2017; Valero et al., 2017).

### 4.3 Resource utilization and hospital economics

The implementation of a selective ICU admission strategy following elective craniotomy has substantial implications for resource utilization and hospital economics. ICU beds represent a limited and cost-intensive resource, with daily costs far exceeding those of standard ward care (Hecht et al., 2014; Valero et al., 2017). In high-income healthcare systems, the average daily cost of an ICU bed ranges from €1200 to €2500 in Europe (Martin et al., 2008.) and up to $3000 to $4500 in the United States (Dasta et al., 2005), compared to €300–€700 per day on a general neurosurgical ward (Norris et al., 1995).

In our study, 82 % of patients were managed safely without ICU admission. If ICU admission had been applied indiscriminately to all elective craniotomy patients, this would have resulted in over 800 additional ICU days during the study period. Assuming average ICU costs of €1500 per day, this equates to potential savings exceeding €1.2 million, not accounting for indirect costs such as staffing, equipment, or opportunity loss. Selective ICU allocation based on individualized risk profiling thus represents a substantial opportunity for cost reduction. Similar findings have been reported by Beauregard et al. (Beauregard and Friedman, 2003), who estimated cost savings of over $4000 per patient using a selective monitoring strategy after craniotomy.

In addition to financial savings, reduced ICU utilization minimizes exposure to ICU-specific complications such as nosocomial infections, delirium, and overtreatment with unnecessary invasive monitoring. Furthermore, it improves ICU bed turnover and allows hospitals to reallocate critical care resources to patients with higher acuity needs.

### 4.4 Limitations

This study has several limitations. First, its retrospective, single-center design may limit generalizability to other institutions with different patient populations, surgical case mixes, or resource availability. Second, some postoperative ICU admissions were influenced by non-medical factors such as bed availability and staffing constraints, which may have introduced bias in the assessment of clinical risk. Third, although a risk score was developed and internally validated, the overall performance of the risk score was moderate (AUC 0.76), which may limit its predictive precision on an individual level. External validation in independent cohorts is needed before broader clinical implementation. Moreover, the score includes two intraoperative variables – surgical time and blood loss – which demonstrated the strongest individual predictive performance based on AUC analysis and are well established in the literature as independent risk factors for ICU admission (Florman et al., 2017; Hanak et al., 2014; Badenes et al., 2017; Neumann et al., 2023). While a purely preoperative model would offer theoretical advantages for early triage, excluding intraoperative parameters would significantly reduce predictive accuracy. Therefore, the score is best interpreted as a pragmatic intraoperative decision-making tool, with final risk estimation completed once key intraoperative thresholds are reached.

While the present analysis focused on clinical and intraoperative variables, we acknowledge that radiological factors – such as lesion size, peritumoral edema or midline shift – may also influence ICU/IMC admission risk. Given the large cohort size and the complexity of imaging data, these variables were not included in the current model but should be investigated in a dedicated study.

## 5 Conclusion

The present study supports the safety and feasibility of a selective ICU admission strategy following elective craniotomy. By identifying key risk factors for unplanned postoperative ICU/IMC admission – including surgical duration, blood loss, advanced age, and intraventricular lesion location – we developed a pragmatic clinical risk score. Despite moderate discriminatory performance (AUC 0.76), the score may serve as a practical tool. Importantly, our data suggest that even complex and infratentorial procedures can be safely managed outside the ICU in the absence of individual risk factors. Adopting a risk-adapted postoperative management strategy holds substantial potential to reduce unnecessary ICU utilization, minimize healthcare costs, and optimize critical care capacity without compromising patient safety.

## Author contributions

Conception and design: Marcus Czabanka, Lina-Elisabeth Qasem, Vincent Prinz, Michael Eibach, Jürgen Konczalla, Ulrich Strouhal, Kai Zacharowski, Christian Grefkes-Hermann

Acquisition of data: Lina-Elisabeth Qasem, Dilara Karakaya, Ali Al-Hilou, Christian Seemann, Felix Corr, Celina Ufken, Frenki Shima

Analysis and interpretation of data: Lina-Elisabeth Qasem, Jan Oros, Stefanos Voglis, Kristin Lucia

Drafting the manuscript: Lina-Elisabeth Qasem, Marcus Czabanka.

All authors reviewed the results and approved the final version of the manuscript.

## Data availability statement

The datasets used and analyzed during the current study are available from the corresponding author on reasonable request.

## Disclosures

The authors have no personal, financial, or institutional interest in any of the drugs, materials, or devices described in this article. This work was not supported by any external funding.

## Declaration of generative AI and AI-assisted technologies in the writing process

During the preparation of this manuscript, the authors used OpenAI's ChatGPT-4.0 to improve the clarity and language of the text. After using this tool, the authors carefully reviewed, edited, and approved all content. The authors take full responsibility for the integrity and accuracy of the final version.

## Declaration of competing interest

The authors declare that they have no known competing financial interests or personal relationships that could have appeared to influence the work reported in this paper.
